# Intake of Meat Proteins Substantially Increased the Relative Abundance of Genus *Lactobacillus* in Rat Feces

**DOI:** 10.1371/journal.pone.0152678

**Published:** 2016-04-04

**Authors:** Yingying Zhu, Xisha Lin, He Li, Yingqiu Li, Xuebin Shi, Fan Zhao, Xinglian Xu, Chunbao Li, Guanghong Zhou

**Affiliations:** Key Laboratory of Meat Processing and Quality Control, MOE; Key Laboratory of Animal Products Processing, MOA; Jiang Synergetic Innovation Center of Meat Processing and Quality Control; Nanjing Agricultural University; Nanjing 210095, P.R. China; University of Illinois at Urbana-Champaign, UNITED STATES

## Abstract

Diet has been shown to have a critical influence on gut bacteria and host health, and high levels of red meat in diet have been shown to increase colonic DNA damage and thus be harmful to gut health. However, previous studies focused more on the effects of meat than of meat proteins. In order to investigate whether intake of meat proteins affects the composition and metabolic activities of gut microbiota, feces were collected from growing rats that were fed with either meat proteins (from beef, pork or fish) or non-meat proteins (casein or soy) for 14 days. The resulting composition of gut microbiota was profiled by sequencing the V4-V5 region of the 16S ribosomal RNA genes and the short chain fatty acids (SCFAs) were analyzed using gas chromatography. The composition of gut microbiota and SCFA levels were significantly different between the five diet groups. At a recommended dose of 20% protein in the diet, meat protein-fed rats had a higher relative abundance of the beneficial genus *Lactobacillus*, but lower levels of SCFAs and SCFA-producing bacteria including *Fusobacterium*, *Bacteroides* and *Prevotella*, compared with the soy protein-fed group. Further work is needed on the regulatory pathways linking dietary protein intake to gut microbiota.

## Introduction

Meat has exerted a crucial role in human evolution and is an important component of a healthy and balanced diet because of its high abundance of proteins, fatty acids, minerals and vitamins. Meat proteins are composed of more balanced essential amino acids than plant proteins and have diverse biological functions [1]. However, the global transition towards an increasing proportion of red meat in human diets may be associated with metabolic disorders [2]. Numerous epidemiological studies have shown that excessive intake of red meat results in the development of cardiovascular disease and colorectal cancer [3–5]. The possible association between red meat intake and colorectal cancer is commonly explained by production of carcinogens during cooking or processing of meat products [6]. Recently, the International Agency for Research on Cancer, a branch of the World Health Organization, issued a report in which red meat and processed meat were listed as carcinogenic agents (http://www.iarc.fr/en/media-centre/pr/2015/pdfs/pr240_E.pdf), but the conclusions of this report have been debated worldwide. However, it is the case that excessive intake of red meat or any other foods may have a detrimental effect on human health, probably inducing some kinds of metabolic disorders. Meat proteins have been distinguished by its richness in all the essential amino acids with no limiting amino acids, but few data are available on the physiological responses of our bodies to different types of dietary protein. A previous study indicated that substitution of red meat by other healthy protein sources such as nut, soy, fish or chicken, decreased the incidence of cardiovascular disease and colorectal cancer [7]. However, the underlying mechanism for this is not clear.

Foods are mainly digested in the stomach and the small intestine, but indigestible food compounds and endogenous proteins secreted in the small intestine enter into the large intestine for microbial fermentation and putrefaction, which shape a diverse gut microbiota [8, 9]. The bacteria residing in the gut are recognized as an essential "organ" and a crucial factor in human physiology and nutrition [10–13]. This organ may protect the host against pathogens and enhance metabolic capabilities [14]. Previous studies focused mostly on the nutritional relevance of dairy and plant proteins to meat proteins [15–17]. Protein level and source (e.g., milk or soy) may affect the intestinal microbial balance [18]. Limited studies have been conducted to investigate the effect of meat, rather than meat proteins, on gut health. [19, 20]. In vitro incubation of cooked beef, chicken or fish meat with human feces led to a significant difference in the numbers of *Bifidobacterium* spp. and *Bacteroides* [21]. It is not known whether short-term dietary intake of different meat proteins affects the composition of gut microbiota and their activities.

In the present study, AIN-93G rat diets were prepared using proteins from beef, casein, fish, pork and soy, and then growing rats were fed these diets at the recommended level of 20% protein for 14 days. The composition of gut bacteria and short-chain fatty acids (SCFAs) in feces were analyzed to compare differences as a response to dietary protein intake.

## Materials and Methods

### Animals and diets

Fifty-five male 4-week-old Sprague-Dawley rats were obtained from a commercial experimental animal center (Zhejiang, China, SCXK9<Zhejiang>2008–00) and reared in a specific pathogen-free facility (SYXK<Jiangsu>2011–0037). The protocol was approved by the Ethical Committee of Experimental Animals of Nanjing Agricultural University. Animals were handled as we previously described [22]. After 7 days acclimatization, animals were divided into five diet groups, fed pork protein, beef protein, fish protein, soy protein or casein (n = 11 each group). The animals were individually housed in plastic cages and given water and food *ad libitum* for 14 days.

The formulation of animal diets referred to the AIN-93 standard for growing rats [23] and diets were prepared as we previously described [22]. Briefly, dietary proteins were extracted from beef *longissimus dorsi* muscle, pork *longissimus dorsi* muscle and fish muscle. Scales, bones, visible fat and connective tissue were removed before protein extraction. These muscles were finely chopped and placed in plastic bags and cooked in a 72°C water bath until the center temperature reached 70°C, and then the cooked samples were chilled, freeze-dried and ground into powder. Casein was obtained from Jiangsu Teluofei, Inc. (Nantong, China) and soy protein was obtained from Linyi Shansong Biological Products Inc. (Linyi, China). Intramuscular fats in meat were removed by extracting for three time in 3 volumes of methylene chloride/methanol mixture (V/V = 2:1). Isoflavones in soy protein were removed by 80% methanol (W/V = 1kg: 6.25L). The protein percents were 87%, 89.25%, 92.48%, 94.18% and 93.42% in casein, soy, fish, pork and beef proteins, and the other nutrients included water, and small amounts of minerals, fat and fibers. More details of the composition of protein powders can be seen in S1 Table. Meanwhile, we detected the composition of amino acids (S1 Fig) and minerals (S2 Table) in different dietary proteins. The AIN-93G mineral mixture (S3 Table) of diet is based on the results of mineral composition of dietary proteins. The diet composition can be seen in Table 1.

**Table 1:** The composition of five formulated diets.

Component (g/kg)	casein	pork	fish	beef	soy
Protein powder^1^	200	185	188	186	195
Cornstarch	397.5	397.5	397.5	397.5	397.5
Dextrinized cornstarch	132	132	132	132	132
Sucrose^2^	100	95.2	92.9	95.2	96.2
Soybean oil	70	70	70	70	70
Fiber	50	50	50	50	50
Mineral mixture^3^	35	35	35	35	35
AIN-93G vitamin mixture^4^	10	10	10	10	10
L-Cystine	3	3	3	3	3
Choline bitartrate	2.5	2.5	2.5	2.5	2.5
Water^5^	0	19.8	19.1	18.8	8.8
Nutritional level					
Energy, Kcal^6^	3706	3706	3706	3706	3706
Total protein, g	177	177	177	177	177
Total fat, g	70	70	70	70	70
Total carbohydrate, g	629.5	629.5	629.5	629.5	629.5
Fiber, g	50	50	50	50	50

^1^ Protein powders contain certain quantities of moisture, minerals and fats/lipids (seen in S1 Table). 174g protein was from protein powder, the actual protein content was 177 g/kg (protein powder and L-cystine) for all the diets.

^2^ Sucrose was applied for the preparation of mineral mixtures. And thus sucrose was balanced to a final content 100g/kg.

^3^ The minerals were balanced by mixing different compounds although protein powders contained different amounts of them. Mineral mixtures were list in S3 Table.

^4^ The formulation of vitamin mixtures as described by Reeves et. al [22].

^5^ Water was added to balanced other nutrients.

^6^ The energy of diets was calculated based on the contents of protein, fats and sugars.

### Sample collection

Fresh feces were collected after rats were fed for 14 days. Normally, the animals excrete feces when they are hung by their tails. The fecal samples were immediately frozen in liquid nitrogen and then stored at −80°C until further analyses.

### Bacterial community analysis

Total microbial DNA was extracted from fecal samples using a commercial stool DNA extraction kit (Qiagen, Germany, No. 51504) as the manufacturer’s protocol. All DNA samples were kept at −20°C until sequencing. The V4-V5 hypervariable region of the 16S ribosomal RNA gene was selected for amplification from DNA samples. The universal primers used were F515 (5′-GTGCCAGCMGCCGCGG-3′) and R907 (5′-CCGTCAATTCMTTTRAGTTT-3′) which also carried an eight-base unique sequence (a so called barcode) for each sample [24]. PCR reactions were run and amplicons sequenced as described previously [22].

### SCFA determination

SCFAs, including acetic, propionic, butyric, isobutyric, isovaleric and valeric acids were detected by gas chromatography (GC) according to a previous protocol [25]. Briefly, 200 mg of a fecal sample were suspended and homogenized in 1 mL ddH_2_O, and then centrifuged (4°C, 13,000 × *g*) for 10 min. Five hundred microliters of the supernatants were mixed with 100 μL of solution in which 0.65 g of crotonic acid was dissolved in 100 mL 25% metaphosphoric acid; crotonic acid was used as an internal standard. The samples were analyzed on a GC system (Thermo Fisher Scientific, USA) and SCFAs were detected with a flame ionization detector under the following conditions: injection volume, 1 μL; oven temperature, 130°C; inlet and outlet temperatures, 180°C; runtime 10 min.

### Bioinformatics and Statistical analysis

Bioinformatics analysis referred to our previous study [22]. Raw fastq files were demultiplexed, quality-filtered using QIIME (version 1.17): (1) the 250 bp reads were truncated at any site receiving an average quality score <20 over a 10 bp sliding window. (2) the truncated reads shorter than 50bp were removed. (3) exact barcode matching was defined that not more than 2 bp mismatching with primer. (4) reads containing ambiguous characters were removed. (5) the sequences that overlap longer than 10 bp were assembled according to their overlap sequence. (6) Reads which could not be assembled were discarded. Operational Taxonomic Units (OTUs) were clustered with 97% similarity cutoff using UPARSE (version 7.1 http://drive5.com/uparse/) and chimeric sequences were identified and removed using UCHIME. The phylogenetic affiliation of each 16S rRNA gene sequence was analyzed by RDP Classifier (http://rdp.cme.msu.edu/) against the silva (SSU119) 16S rRNA database using confidence threshold of 70%. Rarefaction analysis and alpha diversities were performed using Mothur. Community diversity was evaluated by Shannon index and Simpson index. Community richness was evaluated by Chao and ACE. The heatmap and clustering analysis was preformed by R package (R 3.0.2).

One-way analysis of variance was performed to evaluate the differences in SCFAs and the relative abundance of fecal bacteria among the five diet-groups. Duncan’s multiple comparison was applied to compare averages between any two groups. Differences were considered significant if p values were < 0.05. All analyses were performed using SAS software (version 9.2).

## Results

### Richness and diversity analyses

We obtained 1,469,231 usable raw reads from 49 fecal samples (S2a Fig), corresponding to 793 OTUs with an average of 371 ± 60 per biological sample at a similarity level of 97% (S2b Fig). Six fecal samples could not be obtained during the course of feeding, including three from the beef protein-fed group, two from the pork protein group and one from the fish protein group. The pork protein group had a greater number of usable raw reads than the soy protein group (p < 0.05), but there was no significant difference in the number of OTUs between any two diet groups. Rarefaction analysis (S2c Fig), Shannon-Wiener diversity (S2d Fig) and Good’s coverage index (99.74% ± 0.07%) indicated that the sequencing methodology was appropriate to evaluate the microbial diversity in the present study. Diet did not affect ACE, Chao, Shannon, Simpson, and Good’s coverage indices for gut bacteria (S4 Table).

### Composition of gut bacteria in feces

**Fig 1:**
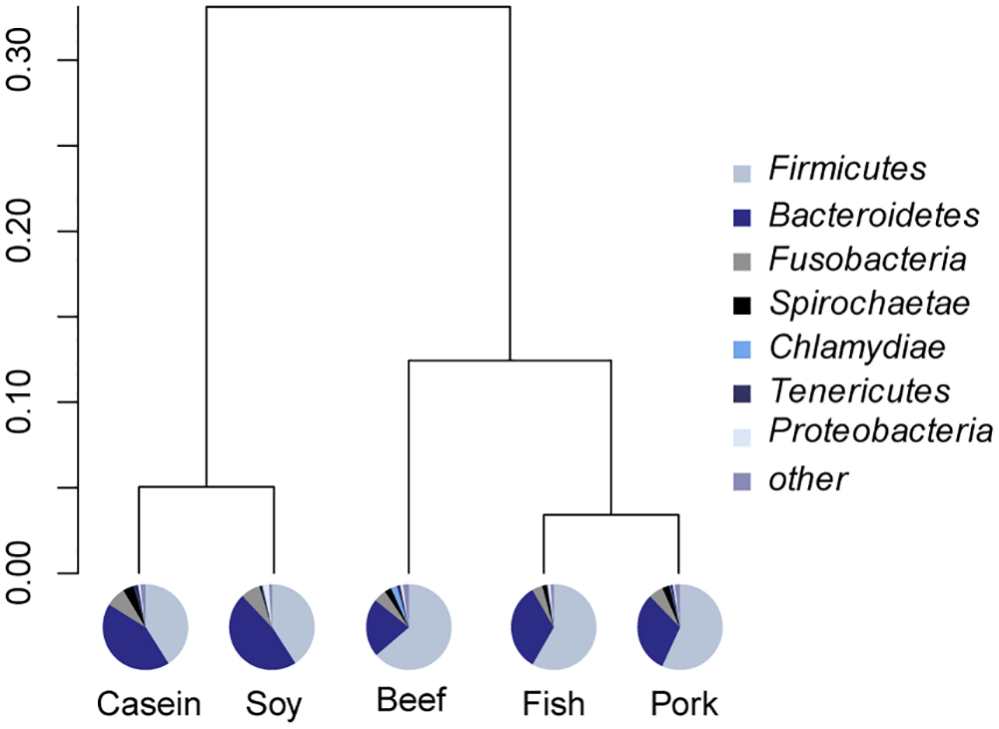
Relative abundance of gut bacteria at the phylum level. Pie charts show the composition of gut bacteria at the phylum level. Bray-Curtis similarity cluster analysis shows that the composition of gut bacteria in feces from the beef, pork and fish protein-fed groups could be separated from those of the casein and soy protein-fed groups.

At the phylum level, Firmicutes and Bacteroidetes were predominant in all samples. Rats fed with proteins from beef, pork and fish had a higher average relative abundance of Firmicutes (p < 0.05, Fig 1), but rats fed with casein and soy protein had a higher abundance of Bacteroidetes (p < 0.01). The relative abundance of Spirochaetae was the highest in the casein group (p < 0.05). Clustering analysis indicated that gut microbiota from the beef, pork and fish groups could be classified into one category and those from the casein and soy protein groups could be considered another category (Fig 1).

**Fig 2:**
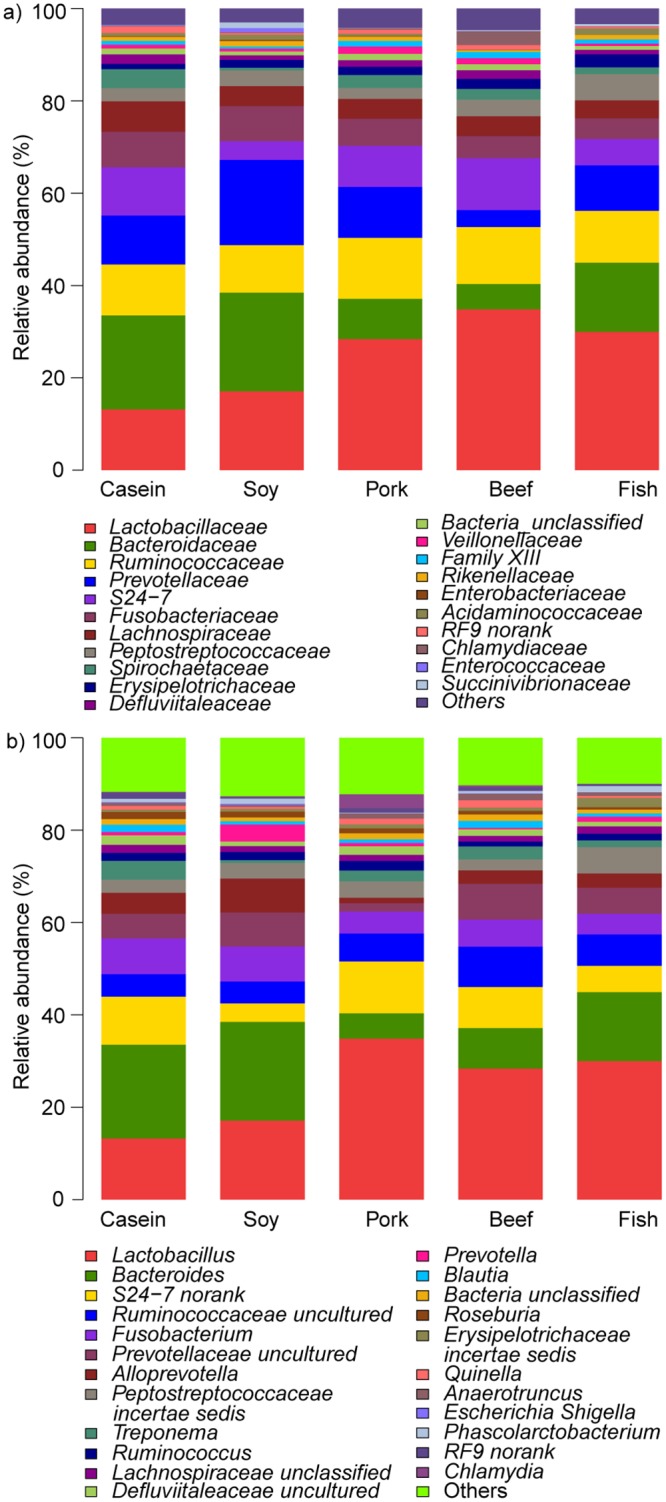
Relative abundance of gut bacteria in rat feces at the family and genus levels. a) At the family level. b) At the genus level.

At the family level, the composition of gut bacteria varied greatly with diet (p < 0.05, Fig 2a). The casein group had the lowest relative abundance of Lactobacillaceae (p < 0.05) but the highest of Lachnospiraceae (p < 0.05), and the soy protein group had the lowest abundance of Prevotellaceae (p < 0.05). However, these two groups showed higher abundances of Bacteroidaceae than the beef, pork and fish protein groups (p < 0.05).

**Fig 3:**
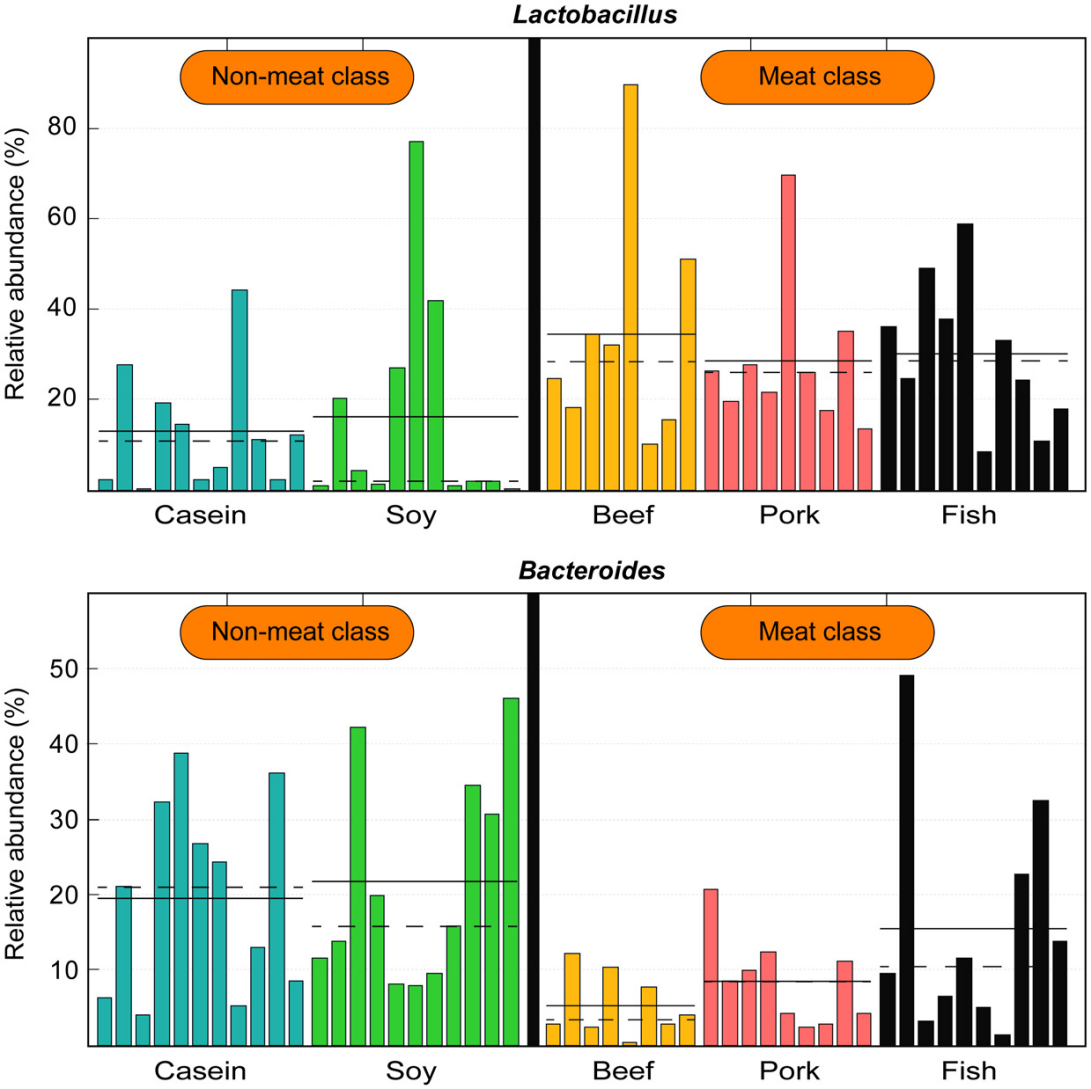
Relative abundance of *Bacteroides* and *Lactobacillus* in different diet groups. The mean and median relative abundances are indicated with solid and dashed lines respectively. Each column represents one biological sample and there are 49 biological samples in total, including 11 from the casein group, 11 from the soy protein group, 8 from the beef protein group, 9 from the pork protein group and 10 from the fish protein group. The samples were classified into "non-meat" (casein and soy protein) and "meat" (beef, pork and fish proteins).

At the genus level, rats fed with pork and beef proteins had higher relative abundances of *Allobaculum* but lower *Lachnospiraceae* uncultured and *Lachnospiraceae* incertae sedis than the casein group (p < 0.05; Fig 2b). *Blautia* was more abundant in the soy, beef and fish protein-fed groups compared to the casein group (p < 0.05). Casein and soy protein groups had higher relative abundances of *Bacteroides* but lower *Lactobacillus* than the other three groups (p < 0.05). No significant difference existed in the relative abundances of *Bacteroides* and *Lactobacillus* between the casein and soy protein groups. If the five diet groups were classified into "meat" (including beef, pork and fish protein groups) and "non-meat" (including casein and soy protein groups), the meat category showed a higher relative abundance of *Lactobacillus* but a lower relative abundance of *Bacteroides* than the non-meat category (p < 0.05; Fig 3).

**Fig 4:**
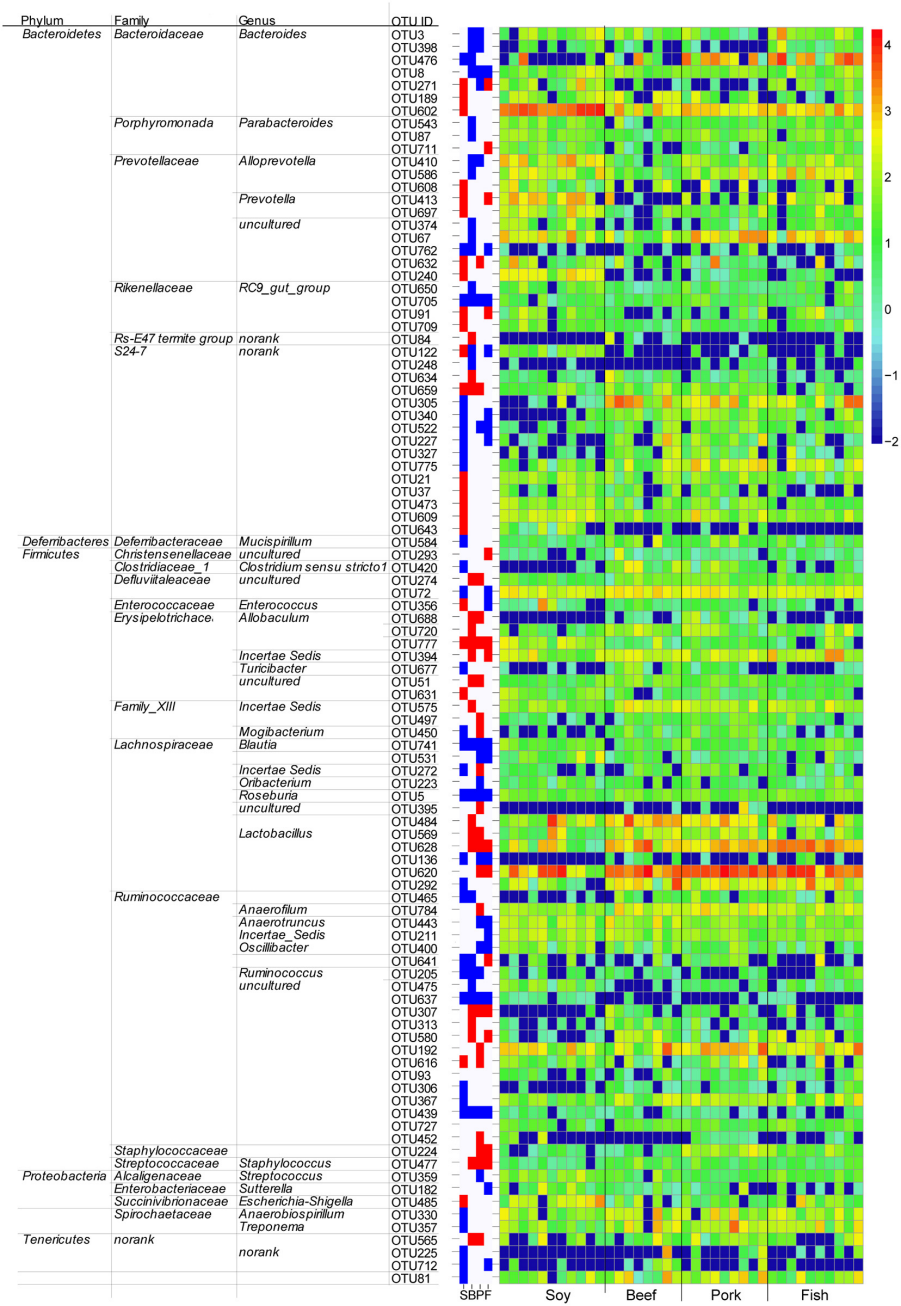
Differences in bacterial communities at the OTU level. The figure includes three parts: 1) The right panel shows the relative abundance (log 10 transformation) of OTUs. Each column represents one biological sample and each row represents one OTU; 2) the middle panel shows the fold-changes of OTUs that changed significantly (p < 0.05) compared to the casein group. Red denotes an increase, blue denotes a decrease. S, soy protein group; B, beef protein group; P, pork protein group; F, fish protein group; 3) the left panel lists significantly changed OTUs and the corresponding phyla, families and genera.

The above observations indicated that gut bacteria in feces differed depending on the dietary proteins. To characterize specific bacteria related to diet, linear discriminant analysis effect size (LefSe) analyses were performed on those OTUs with relative abundances >0.1% in any given group. The overall profiles of gut bacteria differed significantly between the casein group and all the other groups (p < 0.05; Fig 4 and S5 Table). Ninety-nine OTUs were significantly different between the casein group and other groups (at least one group). Ninety-three of these OTUs belong to the phyla Firmicutes and Bacteroidetes. The beef and soy protein-fed groups had a much lower abundance of OTU476 (genus *Bacteroides*) than the casein group (averages 11.08%, 0.89% and 1.28% for casein, beef and soy protein groups, respectively; p < 0.001), but no significant difference was observed among the casein, pork and fish protein groups. OTU628 (genus *Lactobacillus*) was higher in three meat protein groups than in the casein and soy groups (averages 6.11%, 3.42%, 8.60%, 1.05% and 1.06% for beef, pork, fish, casein and soy protein groups, respectively; p < 0.01). OTU620 (genus *Lactobacillus*) was more abundant in the fish and pork protein groups than in the casein group (averages 17.23%, 21.25% and 4.94% for fish, pork and casein groups, respectively; p < 0.01).

### SCFA profiling

In general, total fatty acids showed significant differences among the five diet groups (p < 0.05; Table 1). Acetic acid accounted for 64.75% to 71.18% of the total fatty acids varying with diet, ranging from 29.29 to 35.81 μmol/g feces. Propionic and butyric acids also had relatively high concentrations, ranging from 6.22 to 7.93 μmol/g feces and from 5.00 to 7.48 μmol/g feces, respectively (Table 2). The type of proteins in the diet significantly affected the SCFA composition in rat feces. The soy protein-fed group had the highest levels of total SCFAs and of individual SCFAs except acetic acid (p < 0.05; Table 2). The casein group had the lowest levels of total SCFAs, acetic acid and isobutyric acid. Of the three meat protein-fed groups, the fish group had the highest level (p < 0.05) of isovaleric acid, while the pork protein group had the highest level of isobutyric acid.

**Table 2:** Effect of the type of dietary protein on SCFA levels (μmol/g, means ± standard deviations).

	Casein	Soy	Beef	Pork	Fish
Total	44.07±5.95^b^	52.40±8.70^a^	49.49±9.06^a^^b^	50.31±3.35^a^^b^	44.05±3.70^b^
Acetic	29.29±4.82^b^	33.93±7.49^a^^b^	35.16±7.96^a^	35.81±3.09^a^	30.89±3.04^a^^b^
Propionic	7.12±1.99 ^a^^b^	7.93±1.4^a^	6.62±1.17^a^^b^	6.75±1.19^a^^b^	6.22±0.60^b^
Butyric	5.44±0.97^b^	7.48 ±0.86^a^	5.64±0.93^b^	5.41±0.95^b^	5.00 ±0.96^b^
Isobutyric	0.33±0.08^c^	0.91±0.09^a^	0.57±0.08^b^	0.81±0.14^a^	0.47±0.16^b^
Isovaleric	0.55±0.09^b^	0.75±0.12^a^	0.44±0.09^c^	0.45±0.08^c^	0.56±0.09^b^
Valeric	1.33±0.18^a^	1.40±0.23^a^	1.05±0.44^b^	1.08±0.13^b^	0.90±0.05^b^

The data were analyzed by one-way analysis of variance and means were compared by Duncan’s multiple comparison.

^a,b,c^ Means with different superscripts differed significantly (p < 0.05).

### Growth performance and food intake

There was no significant difference in body weight between any two groups on day 0 (Table 3). After 14 days feeding, the fish protein group showed the highest body weight and body weight gain; the lowest values were found for the soy protein group (p < 0.05). Meanwhile, the soy protein group had the lowest food intake in five groups numerically, although there was no significant difference between any two groups (p>0.05).

**Table 3:** Effect of the type of dietary protein on growth performance and food intake of rats.

Group	Casein	Soy	Beef	Pork	Fish
Body weight (0d, g)	167±15^a^	168±12^a^	169±15^a^	168±15^a^	171±12^a^
Body weight (14d, g)	329±22^a^^b^	298±20^c^	318±29^b^^c^	320±22^b^	343±24^a^
Body weight gain (g)	162±13^a^^b^	130±13^c^	149±17^b^	152±22^b^	172±23^a^
Food intake (g/day)	20.5±1.2^a^	17.5±0.8^a^	20±1.3^a^	20.1±1.4^a^	20±1.2^a^

The data were analyzed by one-way analysis of variance and means were compared by Duncan’s multiple comparison.

^a,b,c^ Means with different superscripts differed significantly (p < 0.05).

## Discussion

The gut has been considered a moving bioreactor that provides undigested food compounds and endogenous compounds for maintaining a highly diverse chemostat culture [9, 10, 26]. It is not surprising that the composition of gut bacteria may be shaped by diet. For example, a high-fat diet decreases the relative abundance of *Bacteroides* and *Bifidobacterium* in feces [27]. However, it was difficult to draw any conclusion whether the diet-induced difference should be attributed to long-term or short-term effects. We showed previously that long-term intake of different meat proteins at the recommended level led to different composition of gut bacteria in the rat caecum [22]. The present study provided further evidence that it was protein source but not feeding time that affected the composition gut bacteria. Fourteen days might be enough for gut microbiota to reach a stable state after a change in diet.

SCFAs are the end products of carbohydrates and proteins fermentation in the large intestine. Butyrate is mainly utilized by enterocytes. The majority of acetate and propionate are utilized by other tissues. Different gut bacteria have different preferences for substrate and produce different SCFAs. The composition of SCFAs varies with dietary carbohydrates, dietary proteins and endogenous proteins. There were substantial undigested fibers, glycans and undigested resistant starch can enter into large intestine for the utilization of gut bacteria [28]. Dietary proteins that are not completely digested and absorbed in the small intestine can also enter into the large intestine and are used by gut bacteria [8, 9]. According to the manufacturer (Linyi Ltd. Co.), there was less than 0.5% crude fiber in soy protein powder, which accounted for 0.00975 g/kg in diet. Normally, daily diet intake of rats in the present study is about 17.5 g, and the amount of fiber from soy protein is less than 0.00975 g. And thus we think that such a small amount of fiber may hardly affect the composition of gut bacteria as compared to other kinds of components in diets. The differences in the levels of SCFAs among dietary groups could mainly be ascribed to dietary proteins and the composition of gut bacteria. There are at least 81 different glycoside hydrolase families in gut bacteria involved in starch and sucrose metabolism, which lacked in the host [29]. For example, *Bacteroides* can use a series of multi-enzyme systems, named the Sus-like systems, to produce SCFAs [30]. Rat feces from the soy protein group had the highest level of total SCFAs. This result indicates that meat proteins intake could reduce the fermentation of non-digested fibers in rat. The composition of amino acids in different dietary proteins may also affect SCFA profiling. In gut bacteria, acetic acid can be produced from glycine, alanine, threonine, glutamate, lysine and aspartate, while butyric acid may be produced from glutamate and lysine, propionic acid from alanine and threonine, and isobutyric and isovaleric acid from valine and leucine [31]. The composition of amino acids in five dietary proteins was significantly different (S2 Fig). These differences maybe course cause different levels of SCFAs. Soy protein contains relatively low levels of threonine, valine, leucine and lysine, but samples from the rats fed with soy protein showed higher levels of propionic, butyric, isobutyric and isovaleric acids than the other diet groups. This could be explained by two factors: (1) the bioavailability of soy protein in the small intestine may be lower than that of casein and meat proteins, resulting in the passage of more undigested proteins and peptides into the large intestine [32]. In addition, soy protein was shown to be able to stimulate epithelial cells in the small intestine to excrete more endogenous proteins [33]. These undigested and endogenous proteins can enter the large intestine for microbial fermentation and thus more SCFAs may be produced. (2) The higher level of SCFAs in the soy protein group may also be associated with the higher relative abundance of *Bacteroides* and *Prevotella* in this group (S6 Table). *Bacteroides* and *Prevotella* have the capability to use a wide range of substrates and are the major propionate and other SCFAs producers [34].

Compared with casein and soy protein diets, the intake of meat proteins was shown to increase the abundance of the genus *Lactobacillus*. Members of this genus have been proposed to be key players in host metabolic homeostasis because they can protect the gut barrier against disruption by pathogens and can reduce inflammation [35–38]. The high abundance of *Lactobacillus* in meat protein groups may be beneficial for the host.

Previous studies have shown that excessive intake of red meat may be associated with a high risk of mortality from colorectal cancer [5, 8], The underlying mechanisms may be as follows [3, 39–41]: (1) nitroso-compounds formed by gut bacteria in the gastrointestinal tract by N-nitrosation of peptide-derived amines or nitrosylation reactions are toxic. (2) Heterocyclic amines formed during high-temperature roasting have cytotoxicity in the gut. (3) The high level of heme iron in red and processed meats can increase the redox level and induce inflammation. (4) An unbalanced composition of gut bacteria characterized by high *Fusobacterium* and *Bacteroides* but low *Lactobacillus*. In the present study, N-nitroso-compounds and heterocyclic amines may not be formed because nitrate or nitrite were not added to the diet formulations and meat samples were cooked at low temperatures. Although heme was shown to affect the composition of gut bacteria, the changes in microbiota did not play a causal role in the observed hyperproliferation and hyperplasia [42]. In the present study, the difference in gut bacteria should be attributed to protein source. Although the iron level in diet was balanced, plant material and animal tissues contain different forms of iron with different bioavailability, which may have a certain influence on the gut bacteria. No significant difference was observed in the relative abundance of *Fusobacterium* among the five diet groups. However, the meat protein groups showed higher levels of *Lactobacillus* but lower *Bacteroides* than the non-meat groups. Therefore, cooking method, dose and feeding period may be the critical factors that should be considered when we evaluate associations between the intake of meat proteins, metabolic disorders and other kinds of cytotoxicity.

In summary, in this short-term study, we fed rats with five different protein types, from casein, soy, beef, pork and fish. The type of dietary proteins had a substantial influence on the composition of gut bacteria and SCFAs in rat feces. The five diet groups could be clustered into two subgroups at the level of the phyla of the observed gut bacteria, "meat class" and "non-meat class". This was in accordance with our long-term results. Meanwhile, the relative abundance of the genus *Lactobacillus* was higher in rats fed protein from the meat class than the non-meat class. Specific bacteria sensitive to dietary proteins might play a critical role in the maintenance of a healthy body. Our findings suggest that the intake of meat proteins at a recommended level may increase *Lactobacillus* compared to casein and soy protein diets and thus may benefit gut health. However, rats fed soy protein had the highest level of SCFAs, accompanied by more SCFA-producing bacteria. The underlying mechanism of the regulation of gut microbiota by dietary protein requires further study.

## Supporting Information

S1 Fig The composition of amino acids in different dietary proteins. (TIF)

S2 Fig Diversity estimation of fecal microbial community. a) The average number of usable raw reads (mean and standard deviation); b) The average number of OTU (mean and standard deviation); c) Rarefaction curves. Each curve represents one rat; d) Shannon—Wiener diversity index curves. Each curve represents one rat; Note: there are totally 49 biological samples, of which 11 from casein group (light blue color), 11 from soy protein group (green color), 8 from beef protein group (yellow color), 9 from pork protein group (pink color) and 10 from fish protein group (black color).
(TIF)

S1 Table The composition of five dietary protein powder (g/100g). (DOC)

S2 Table Minerals content of the protein powder. (DOC)

S3 Table Composition levels of mineral premix. (DOC)

S4 Table Richness and diversity indexes relative to each sample (OTUs at 97% similarity). (DOC)

S5 Table The differentially fecal bacterial communities between casein group and any other protein group on OTU level using LEfSe. (DOC)

S6 Table Correlation of fecal bacteria with SCFAs concentration. (DOC)
