# Supplementary figures and images for: Intake of Meat Proteins Substantially Increased the Relative Abundance of Genus Lactobacillus in Rat Feces
Source: PLoS One. 2016 Apr 4;11(4):e0152678. doi: 10.1371/journal.pone.0152678 (PMC4820228; doi:10.1371/journal.pone.0152678)

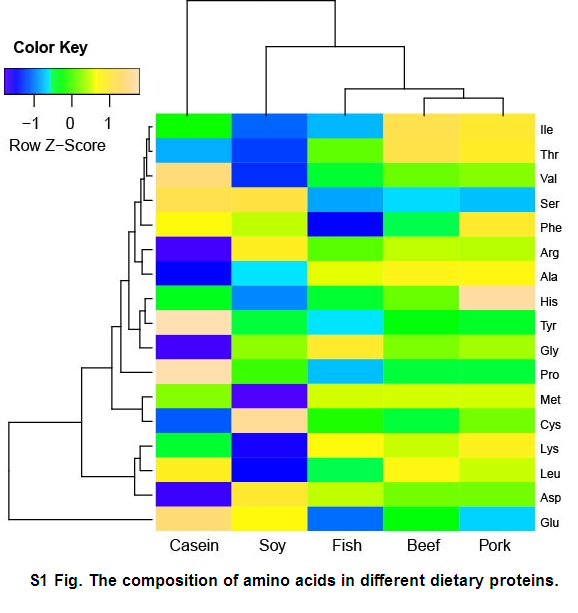

Supplement: S1 Fig — (TIF) [file pone.0152678.s001.TIF]

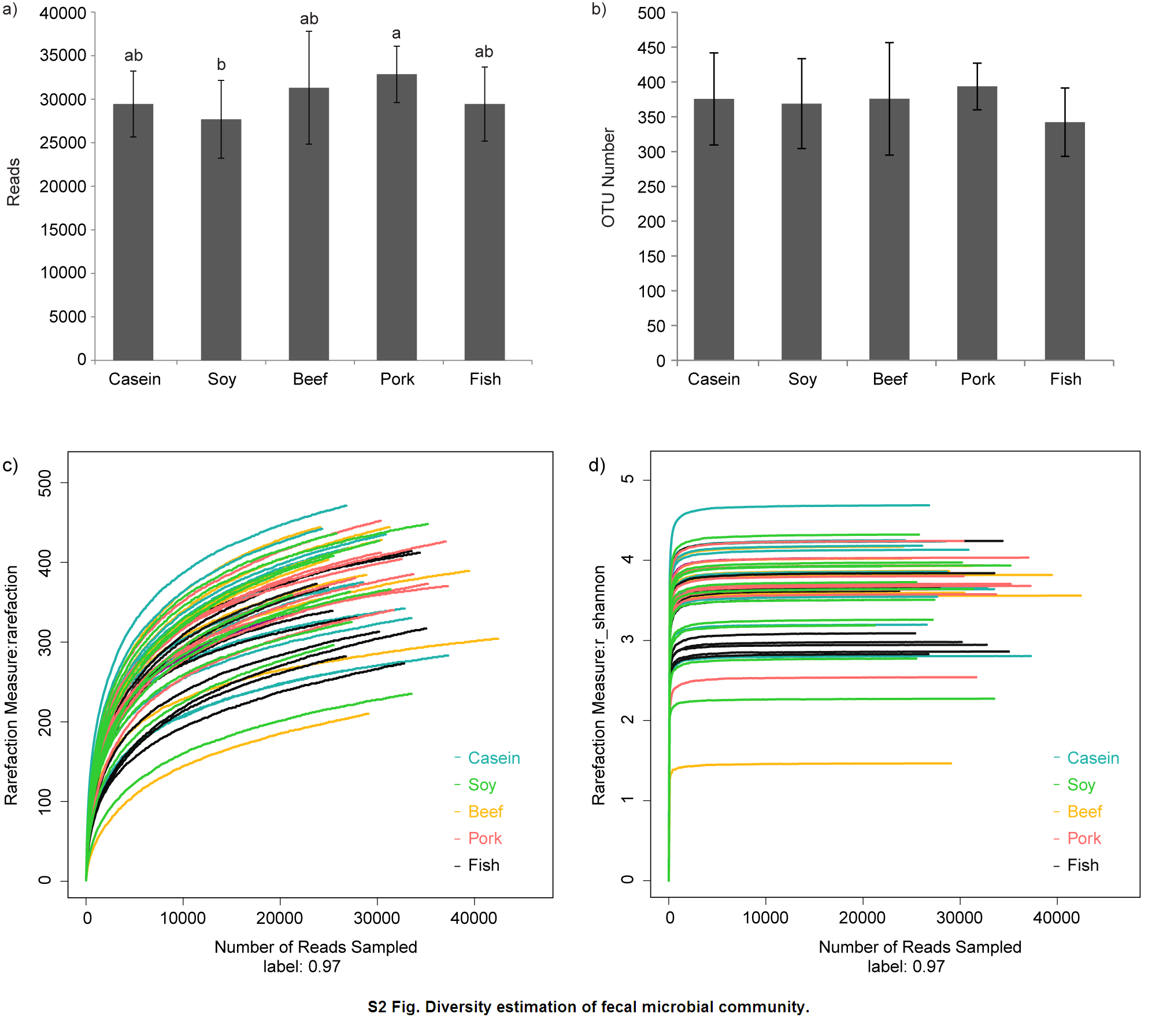

Supplement: S2 Fig — a) The average number of usable raw reads (mean and standard deviation); b) The average number of OTU (mean and standard deviation); c) Rarefaction curves. Each curve represents one rat; d) Shannon—Wiener diversity index curves. Each curve represents one rat; Note: there are totally 49 biological samples, of which 11 from casein group (light blue color), 11 from soy protein group (green color), 8 from beef protein group (yellow color), 9 from pork protein group (pink color) and 10 from fish protein group (black color). (TIF) [file pone.0152678.s002.TIF]
