# Supplementary material for: Intake of Meat Proteins Substantially Increased the Relative Abundance of Genus Lactobacillus in Rat Feces
Source: PLoS One. 2016 Apr 4;11(4):e0152678. doi: 10.1371/journal.pone.0152678 (PMC4820228; doi:10.1371/journal.pone.0152678)
Supplement: S1 Table — (DOC) [file pone.0152678.s003.doc]

**S1** **Table The composition of five dietary protein** powder (g/100g)

|  | Casein | Soy | Fish | Pork | Beef |
| --- | --- | --- | --- | --- | --- |
| Moisture | 10 | 5.69±0.02 | 5.97±0.07 | 4.34±0.15 | 4.95±0.04 |
| Crude ash | 1.9 | 3.96±0.02 | 4.25±0.04 | 3.59±0.07 | 2.88±0.07 |
| Crude fat | 1.1 | 0.43±0.02 | 0.45±0.02 | 0.30±0.02 | 0.40±0.03 |
| Crude protein | 87 | 89.25±0.2 | 92.48±0.1 | 94.18±0.1 | 93.42±0.4 |
