# Supplementary material for: Intake of Meat Proteins Substantially Increased the Relative Abundance of Genus Lactobacillus in Rat Feces
Source: PLoS One. 2016 Apr 4;11(4):e0152678. doi: 10.1371/journal.pone.0152678 (PMC4820228; doi:10.1371/journal.pone.0152678)
Supplement: S2 Table — (DOC) [file pone.0152678.s004.doc]

**S2** **Table** Minerals content of the protein powder

|  | Minerals contents（mg/Kg） | | | | | |
| --- | --- | --- | --- | --- | --- | --- |
|  | Casein | Soy | Fish | Pork | Beef |  |
| Ca | 1614.4±117.3^a^ | 1125.0±151.1^b^ | 1090.8±159.9^bc^ | 914.4±63.4^c^ | 972.9±79.8^bc^ |  |
| Cu | 2.9±0.2^c^ | 17.1±1.3^a^ | 5.0±1.4^b^ | 4.9±0.9^b^ | 5.3±1.3^b^ |  |
| Fe | 70.6±4.6^b^ | 164.6±5.5^a^ | 11.1±2.6^d^ | 35.6±5.1^c^ | 40.9±3.7^c^ |  |
| K | 500.8±8.5^e^ | 1352.5±242.6^d^ | 12824.8±88.5^a^ | 11040.5±122.4^b^ | 8852.2±117.6^c^ |  |
| Mg | 299.4±24.7^e^ | 582.0±53.0^d^ | 1580.5±16.3^a^ | 1348.4±23.9^b^ | 1171.2±15.9^c^ |  |
| Mn | 0.6±0.1^b^ | 8.3±2.4^a^ | 0.8±0.4^b^ | 0.7±0.4^b^ | 0.1±0.1^b^ |  |
| Na | 5029.8±73.1^b^ | 11070.9±1043.5^a^ | 2148.1±89.2^c^ | 2439.1±46.9^c^ | 2156.4±136.8^c^ |  |
| P | 6353.0±60.6^d^ | 7949.1±559.8^cd^ | 8242.5±138.5^bc^ | 8578.6±209.9^b^ | 6433.7±58.9^d^ |  |
| Se | 0.5±0.1^c^ | 0.1±0.0^c^ | 8.2±1.9^a^ | 7.2±1.9^a^ | 3.8±0.9^b^ |  |
| Zn | 44.2±1.8^e^ | 57.0±9.6^cd^ | 65.4±9.9^c^ | 88.7±14.6^b^ | 171.3±5.3^a^ |  |

Note: The different superscript lowercase letters in the same line showed difference (*P*<0.05).
