# Supplementary material for: Intake of Meat Proteins Substantially Increased the Relative Abundance of Genus Lactobacillus in Rat Feces
Source: PLoS One. 2016 Apr 4;11(4):e0152678. doi: 10.1371/journal.pone.0152678 (PMC4820228; doi:10.1371/journal.pone.0152678)
Supplement: S3 Table — (DOC) [file pone.0152678.s005.doc]

**S3 Table Composition level**s of mineral premix

| g/Kg | Casein | Soy | Fish | Pork | Beef |
| --- | --- | --- | --- | --- | --- |
| CaCO_3_ (Ca 40.04%) | 357.00 | 347.87 | 242.62 | 277.02 | 268.34 |
| KH_2_PO_3_ (P 22.76%, K 28.73%) | 196.00 | 255.28 | 40.06 | 116.56 | 118.07 |
| CaHPO_3_ (Ca 23.29%, P 8.00%) | 0.00 | 0.00 | 182.71 | 131.51 | 141.53 |
| K_3_C_6_H_5_O_7_ (K 36.16%) | 70.78 | 0.00 | 0.00 | 0.00 | 0.00 |
| NaCl (Na 39.34%) | 74.00 | 0.00 | 45.35 | 48.17 | 45.55 |
| K_2_SO_4_ (S 18.39% K 44.87%) | 46.60 | 46.60 | 46.60 | 46.61 | 46.60 |
| MgO (Mg 60.32%) | 24.00 | 17.96 | 9.85 | 14.72 | 13.70 |
| FeC_6_H_5_O_7_ (Fe 16.5%) | 6.06 | 0.00 | 5.65 | 5.13 | 4.95 |
| ZnCO_3_ (Zn 52.14%) | 1.65 | 1.34 | 1.37 | 1.30 | 0.27 |
| MnCO_3_ (Mn 47.79%) | 0.63 | 0.49 | 0.64 | 0.64 | 0.65 |
| CuCO_3_ (Cu 57.47%) | 0.30 | 0.14 | 0.29 | 0.29 | 0.29 |
| KIO_3_ (I 59.3%) | 0.01 | 0.01 | 0.01 | 0.01 | 0.01 |
| Na2SeO_3_ (Se 41.79%) | 0.01025 | 0.02 | 0.00 | 0.00 | 0.00 |
| (NH_4_)_6_Mo_7_O_24_ (Mo 54.34%) | 0.00795 | 0.00795 | 0.00795 | 0.00795 | 0.00795 |
| Na_2_SiO_3_·5H_2_O (Si 9.88%) | 1.45 | 1.45 | 1.45 | 1.45 | 1.45 |
| CrK(SO_4_)_2_·12H_2_O (Cr 10.42%) | 0.275 | 0.275 | 0.275 | 0.275 | 0.275 |
| LiCl (Li 16.38%) | 0.0174 | 0.0174 | 0.0174 | 0.0174 | 0.0174 |
| H_3_BO_3_ (B 17.5%) | 0.0815 | 0.0815 | 0.0815 | 0.0815 | 0.0815 |
| NaF (F 45.24%) | 0.0635 | 0.0635 | 0.0635 | 0.0635 | 0.0635 |
| NiCO_3_ (Ni 45%) | 0.0318 | 0.0318 | 0.0318 | 0.0318 | 0.0318 |
| NH_4_VO_3_ (V 43.55%) | 0.0066 | 0.0066 | 0.0066 | 0.0066 | 0.0066 |
| Sucrose | 221.026 | 328.4 | 422.9 | 356.1 | 358.1 |
| Total | 1000.00 | 1000.00 | 1000.00 | 1000.00 | 1000.00 |

Notes: to ensure each mineral content was consistent in five diets, the minerals premixs were prepared according to the mineral content of the protein powder.
