# Supplementary material for: Intake of Meat Proteins Substantially Increased the Relative Abundance of Genus Lactobacillus in Rat Feces
Source: PLoS One. 2016 Apr 4;11(4):e0152678. doi: 10.1371/journal.pone.0152678 (PMC4820228; doi:10.1371/journal.pone.0152678)
Supplement: S4 Table — (DOC) [file pone.0152678.s006.doc]

**S4 Table Richness and diversity indexes relative to each sample (OTUs at 97% similarity)**

| Sample ID | Reads | 0.97 | | | | | |
| --- | --- | --- | --- | --- | --- | --- | --- |
|  |  | OTU | ace | chao | coverage | shannon | simpson |
| Casein1 | 28525 | 375 | 431 | 451 | 99.8% | 4.24 | 0.0309 |
|  |  |  | (409,466) | (416,515) |  | (4.22,4.25) | (0.0301,0.0316) |
| Casein10 | 33498 | 330 | 392 | 418 | 99.8% | 3.64 | 0.0602 |
|  |  |  | (368,432) | (377,494) |  | (3.62,3.66) | (0.0591,0.0614) |
| Casein11 | 24311 | 442 | 511 | 508 | 99.7% | 4.25 | 0.0385 |
|  |  |  | (487,548) | (480,556) |  | (4.23,4.27) | (0.0372,0.0397) |
| Casein2 | 37283 | 283 | 332 | 331 | 99.8% | 2.8 | 0.1369 |
|  |  |  | (312,367) | (308,377) |  | (2.79,2.82) | (0.1349,0.1388) |
| Casein3 | 26559 | 264 | 319 | 314 | 99.8% | 3.2 | 0.1029 |
|  |  |  | (297,357) | (290,361) |  | (3.17,3.22) | (0.1005,0.1053) |
| Casein4 | 28612 | 401 | 467 | 496 | 99.7% | 3.86 | 0.0616 |
|  |  |  | (443,505) | (455,570) |  | (3.84,3.88) | (0.0601,0.0631) |
| Casein5 | 27620 | 371 | 444 | 476 | 99.7% | 3.55 | 0.0713 |
|  |  |  | (418,484) | (431,555) |  | (3.52,3.57) | (0.0698,0.0728) |
| Casein6 | 27067 | 418 | 490 | 486 | 99.7% | 3.7 | 0.0856 |
|  |  |  | (465,528) | (457,537) |  | (3.68,3.73) | (0.0832,0.088) |
| Casein7 | 26798 | 471 | 533 | 531 | 99.7% | 4.69 | 0.0193 |
|  |  |  | (511,568) | (505,576) |  | (4.67,4.7) | (0.0188,0.0198) |
| Casein8 | 32807 | 342 | 382 | 381 | 99.8% | 3.67 | 0.0924 |
|  |  |  | (365,412) | (362,420) |  | (3.65,3.69) | (0.09,0.0947) |
| Casein9 | 30852 | 435 | 502 | 524 | 99.7% | 4.13 | 0.0366 |
|  |  |  | (478,539) | (486,590) |  | (4.11,4.15) | (0.0358,0.0374) |
| Soy1 | 25761 | 436 | 497 | 510 | 99.7% | 4.32 | 0.0299 |
|  |  |  | (475,531) | (478,565) |  | (4.3,4.34) | (0.0291,0.0306) |
| Soy10 | 21894 | 395 | 482 | 472 | 99.6% | 3.92 | 0.0545 |
|  |  |  | (452,526) | (440,526) |  | (3.9,3.95) | (0.0529,0.0561) |
| Soy11 | 27350 | 325 | 385 | 436 | 99.7% | 3.51 | 0.0904 |
|  |  |  | (362,423) | (385,533) |  | (3.48,3.53) | (0.0881,0.0927) |
| Soy2 | 31348 | 366 | 428 | 435 | 99.8% | 3.64 | 0.0756 |
|  |  |  | (405,465) | (404,492) |  | (3.62,3.66) | (0.0739,0.0773) |
| Soy3 | 35186 | 448 | 502 | 507 | 99.8% | 3.94 | 0.0659 |
|  |  |  | (482,534) | (481,554) |  | (3.92,3.96) | (0.0641,0.0676) |
| Soy4 | 33513 | 235 | 307 | 306 | 99.8% | 2.27 | 0.1899 |
|  |  |  | (279,352) | (273,367) |  | (2.25,2.29) | (0.1873,0.1925) |
| Soy5 | 21211 | 359 | 436 | 457 | 99.6% | 3.19 | 0.1457 |
|  |  |  | (409,478) | (416,528) |  | (3.16,3.22) | (0.1416,0.1499) |
| Soy6 | 30183 | 427 | 508 | 522 | 99.7% | 3.97 | 0.0457 |
|  |  |  | (480,551) | (483,590) |  | (3.95,3.99) | (0.0446,0.0467) |
| Soy7 | 27179 | 363 | 446 | 441 | 99.7% | 3.26 | 0.1175 |
|  |  |  | (417,491) | (408,497) |  | (3.23,3.28) | (0.1148,0.1202) |
| Soy8 | 25484 | 408 | 481 | 468 | 99.7% | 3.73 | 0.0896 |
|  |  |  | (456,520) | (443,512) |  | (3.7,3.75) | (0.0869,0.0923) |
| Soy9 | 25483 | 296 | 387 | 391 | 99.7% | 2.77 | 0.1806 |
|  |  |  | (354,438) | (350,463) |  | (2.75,2.8) | (0.1768,0.1844) |
| Beef1 | 39448 | 389 | 446 | 444 | 99.8% | 3.82 | 0.0651 |
|  |  |  | (424,481) | (419,491) |  | (3.8,3.84) | (0.0637,0.0666) |
| Beef10 | 28853 | 384 | 440 | 431 | 99.8% | 3.87 | 0.0549 |
|  |  |  | (419,474) | (410,471) |  | (3.85,3.89) | (0.0536,0.0561) |
| Beef11 | 31247 | 444 | 519 | 527 | 99.7% | 3.93 | 0.0594 |
|  |  |  | (493,558) | (493,586) |  | (3.91,3.95) | (0.0578,0.061) |
| Beef2 | 42424 | 304 | 347 | 338 | 99.9% | 3.56 | 0.0639 |
|  |  |  | (329,379) | (321,373) |  | (3.54,3.58) | (0.0627,0.065) |
| Beef3 | 29063 | 210 | 342 | 352 | 99.7% | 1.47 | 0.4352 |
|  |  |  | (304,394) | (285,479) |  | (1.44,1.49) | (0.4297,0.4406) |
| Beef6 | 24122 | 444 | 506 | 513 | 99.6% | 4.17 | 0.0396 |
|  |  |  | (484,540） | (484,563) |  | (4.15,4.19) | (0.0385,0.0407) |
| Beef7 | 24948 | 403 | 493 | 490 | 99.6% | 4.02 | 0.0453 |
|  |  |  | (463,539） | (455,551) |  | (4,4.04) | (0.044,0.0465) |
| Beef8 | 30445 | 428 | 506 | 510 | 99.7% | 3.59 | 0.0977 |
|  |  |  | (479,547) | (476,567) |  | (3.57,3.62) | (0.0954,0.1) |
| Pork1 | 37247 | 370 | 404 | 410 | 99.9% | 3.68 | 0.0728 |
|  |  |  | (390,430) | (390,450) |  | (3.66,3.7) | (0.0714,0.0743) |
| Pork10 | 37034 | 426 | 509 | 545 | 99.8% | 4.03 | 0.0517 |
|  |  |  | (480,554) | (494,633) |  | (4.01,4.05) | (0.0505,0.0529) |
| Pork2 | 35197 | 373 | 438 | 444 | 99.8% | 3.71 | 0.0659 |
|  |  |  | (414,476) | (413,500) |  | (3.69,3.72) | (0.0643,0.0674) |
| Pork3 | 32487 | 404 | 470 | 488 | 99.8% | 3.94 | 0.0484 |
|  |  |  | (446,508) | (451,555) |  | (3.92,3.96) | (0.0474,0.0495) |
| Pork4 | 31660 | 340 | 405 | 413 | 99.8% | 2.54 | 0.2792 |
|  |  |  | (381,443) | (381,471) |  | (2.51,2.57) | (0.2738,0.2845) |
| Pork6 | 33703 | 385 | 441 | 454 | 99.8% | 3.58 | 0.075 |
|  |  |  | (420,475) | (424,509) |  | (3.56,3.6) | (0.0733,0.0766) |
| Pork7 | 30339 | 452 | 540 | 595 | 99.7% | 4.24 | 0.0345 |
|  |  |  | (510,585) | (536,695) |  | (4.22,4.26) | (0.0336,0.0353) |
| Pork8 | 27704 | 380 | 444 | 447 | 99.7% | 3.64 | 0.0874 |
|  |  |  | (421,481) | (418,498) |  | (3.61,3.66) | (0.085,0.0899) |
| Pork9 | 30356 | 412 | 478 | 471 | 99.7% | 3.8 | 0.0582 |
|  |  |  | (454,515) | (446,515) |  | (3.78,3.82) | (0.0567,0.0596) |
| Fish1 | 24888 | 369 | 445 | 470 | 99.7% | 3.51 | 0.0739 |
|  |  |  | (418,486) | (427,545) |  | (3.48,3.53) | (0.0722,0.0757) |
| Fish10 | 26741 | 282 | 346 | 347 | 99.7% | 2.83 | 0.1474 |
|  |  |  | (322,384) | (318,401) |  | (2.81,2.85) | (0.1443,0.1505) |
| Fish11 | 25368 | 339 | 379 | 368 | 99.8% | 3.09 | 0.1654 |
|  |  |  | (363,406) | (354,395) |  | (3.06,3.12) | (0.1612,0.1695) |
| Fish3 | 35020 | 317 | 407 | 413 | 99.8% | 2.86 | 0.1351 |
|  |  |  | (375,457) | (372,484) |  | (2.84,2.88) | (0.1328,0.1374) |
| Fish4 | 30169 | 313 | 379 | 388 | 99.8% | 2.98 | 0.1268 |
|  |  |  | (354,419) | (354,450) |  | (2.96,3) | (0.1245,0.129) |
| Fish5 | 34363 | 412 | 467 | 470 | 99.8% | 4.24 | 0.0331 |
|  |  |  | (447,500) | (444,517) |  | (4.23,4.26) | (0.0323,0.0338) |
| Fish6 | 32740 | 273 | 355 | 365 | 99.8% | 2.95 | 0.1217 |
|  |  |  | (324,405) | (324,439) |  | (2.93,2.96) | (0.1197,0.1238) |
| Fish7 | 33512 | 414 | 480 | 471 | 99.8% | 3.84 | 0.0486 |
|  |  |  | (456,516) | (446,514) |  | (3.82,3.86) | (0.0477,0.0494) |
| Fish8 | 27892 | 331 | 372 | 371 | 99.8% | 3.65 | 0.087 |
|  |  |  | (355,401) | (351,409) |  | (3.63,3.68) | (0.0847,0.0894) |
| Fish9 | 23737 | 373 | 444 | 438 | 99.7% | 3.62 | 0.0679 |
|  |  |  | (419,484) | (410,487) |  | (3.6,3.64) | (0.0664,0.0694) |
