# Supplementary material for: Intake of Meat Proteins Substantially Increased the Relative Abundance of Genus Lactobacillus in Rat Feces
Source: PLoS One. 2016 Apr 4;11(4):e0152678. doi: 10.1371/journal.pone.0152678 (PMC4820228; doi:10.1371/journal.pone.0152678)
Supplement: S5 Table — (DOC) [file pone.0152678.s007.doc]

**S5** **Table The differentially fecal bacterial communities between casein group and any other protein group on OTU level using LEfSe.**

| OTU ID | Phylum | Family | Genus | Compared group | LDA Scorce (log10) | p value |
| --- | --- | --- | --- | --- | --- | --- |
| OTU122 | Bacteroidetes | S24-7 | *norank* | Beef | -2.606 | <0.05 |
| OTU205 | Firmicutes | Ruminococcaceae | *uncultured* | Beef | -2.880 | <0.01 |
| OTU248 | Bacteroidetes | S24-7 | *norank* | Beef | -2.931 | <0.05 |
| OTU3 | Bacteroidetes | Bacteroidaceae | *Bacteroides* | Beef | -3.479 | <0.05 |
| OTU374 | Bacteroidetes | Prevotellaceae | *uncultured* | Beef | -3.134 | <0.01 |
| OTU398 | Bacteroidetes | Bacteroidaceae | *Bacteroides* | Beef | -2.719 | <0.05 |
| OTU410 | Bacteroidetes | Prevotellaceae | *Alloprevotella* | Beef | -3.885 | <0.001 |
| OTU439 | Firmicutes | Ruminococcaceae |  | Beef | -3.360 | <0.001 |
| OTU465 | Firmicutes | Ruminococcaceae | *Anaerofilum* | Beef | -3.176 | <0.05 |
| OTU475 | Firmicutes | Ruminococcaceae | *uncultured* | Beef | -2.626 | <0.001 |
| OTU476 | Bacteroidetes | Bacteroidaceae | *Bacteroides* | Beef | -4.609 | <0.01 |
| OTU5 | Firmicutes | Lachnospiraceae | *uncultured* | Beef | -3.194 | <0.05 |
| OTU543 | Bacteroidetes | Porphyromonadaceae | *Parabacteroides* | Beef | -2.629 | <0.05 |
| OTU586 | Bacteroidetes | Prevotellaceae | *Alloprevotella* | Beef | -3.454 | <0.05 |
| OTU637 | Firmicutes | Ruminococcaceae | *uncultured* | Beef | -2.897 | <0.01 |
| OTU641 | Firmicutes | Ruminococcaceae | *Ruminococcus* | Beef | -2.638 | <0.05 |
| OTU650 | Bacteroidetes | Rikenellaceae | *RC9_gut_group* | Beef | -2.600 | <0.05 |
| OTU67 | Bacteroidetes | Prevotellaceae | *uncultured* | Beef | -3.585 | <0.05 |
| OTU705 | Bacteroidetes | Rikenellaceae | *RC9_gut_group* | Beef | -2.923 | <0.01 |
| OTU741 | Firmicutes | Lachnospiraceae | *Blautia* | Beef | -3.185 | <0.01 |
| OTU762 | Bacteroidetes | Prevotellaceae | *uncultured* | Beef | -3.075 | <0.05 |
| OTU8 | Bacteroidetes | Bacteroidaceae | *Bacteroides* | Beef | -3.242 | <0.05 |
| OTU87 | Bacteroidetes | Porphyromonadaceae | *Parabacteroides* | Beef | -3.010 | <0.01 |
| OTU274 | Firmicutes | Defluviitaleaceae | *uncultured* | Beef | 2.815 | <0.05 |
| OTU307 | Firmicutes | Ruminococcaceae | *uncultured* | Beef | 2.875 | <0.05 |
| OTU313 | Firmicutes | Ruminococcaceae | *uncultured* | Beef | 2.949 | <0.001 |
| OTU394 | Firmicutes | Erysipelotrichaceae | *Incertae_Sedis* | Beef | 3.309 | <0.05 |
| OTU477 | Firmicutes | Streptococcaceae | *Streptococcus* | Beef | 2.743 | <0.01 |
| OTU484 | Firmicutes | Lactobacillaceae | *Lactobacillus* | Beef | 4.421 | <0.01 |
| OTU51 | Firmicutes | Erysipelotrichaceae | *uncultured* | Beef | 2.937 | <0.05 |
| OTU565 | Tenericutes | norank | *norank* | Beef | 3.203 | <0.05 |
| OTU569 | Firmicutes | Lactobacillaceae | *Lactobacillus* | Beef | 3.391 | <0.01 |
| OTU575 | Firmicutes | Family_XIII | *Incertae_Sedis* | Beef | 3.497 | <0.05 |
| OTU580 | Firmicutes | Ruminococcaceae | *uncultured* | Beef | 3.444 | <0.01 |
| OTU628 | Firmicutes | Lactobacillaceae | *Lactobacillus* | Beef | 4.370 | <0.05 |
| OTU634 | Bacteroidetes | S24-7 | *norank* | Beef | 3.101 | <0.05 |
| OTU659 | Bacteroidetes | S24-7 | *norank* | Beef | 3.110 | <0.01 |
| OTU688 | Firmicutes | Erysipelotrichaceae | *Allobaculum* | Beef | 3.358 | <0.01 |
| OTU720 | Firmicutes | Erysipelotrichaceae | *Allobaculum* | Beef | 3.314 | <0.05 |
| OTU777 | Firmicutes | Erysipelotrichaceae | *Allobaculum* | Beef | 2.924 | <0.01 |
| OTU84 | Bacteroidetes | Rs-E47_termite_group | *norank* | Beef | 2.838 | <0.05 |
| OTU122 | Bacteroidetes | S24-7 | *norank* | Chicken | -3.268 | <0.05 |
| OTU136 | Firmicutes | Lactobacillaceae | *Lactobacillus* | Chicken | -3.180 | <0.001 |
| OTU205 | Firmicutes | Ruminococcaceae | *uncultured* | Chicken | -3.010 | <0.05 |
| OTU223 | Firmicutes | Lachnospiraceae | *Roseburia* | Chicken | -3.279 | <0.05 |
| OTU225 | Tenericutes | norank | *norank* | Chicken | -3.583 | <0.05 |
| OTU3 | Bacteroidetes | Bacteroidaceae | *Bacteroides* | Chicken | -3.554 | <0.01 |
| OTU305 | Bacteroidetes | S24-7 | *norank* | Chicken | -4.316 | <0.05 |
| OTU340 | Bacteroidetes | S24-7 | *norank* | Chicken | -3.360 | <0.01 |
| OTU398 | Bacteroidetes | Bacteroidaceae | *Bacteroides* | Chicken | -3.051 | <0.05 |
| OTU413 | Bacteroidetes | Prevotellaceae | *Prevotella* | Chicken | -3.008 | <0.05 |
| OTU439 | Firmicutes | Ruminococcaceae |  | Chicken | -3.465 | <0.001 |
| OTU465 | Firmicutes | Ruminococcaceae | *Anaerofilum* | Chicken | -3.277 | <0.05 |
| OTU5 | Firmicutes | Lachnospiraceae | *uncultured* | Chicken | -3.287 | <0.01 |
| OTU522 | Bacteroidetes | S24-7 | *norank* | Chicken | -3.166 | <0.01 |
| OTU637 | Firmicutes | Ruminococcaceae | *uncultured* | Chicken | -3.495 | <0.001 |
| OTU705 | Bacteroidetes | Rikenellaceae | *RC9_gut_group* | Chicken | -3.249 | <0.05 |
| OTU712 | Tenericutes | norank | *norank* | Chicken | -3.461 | <0.01 |
| OTU72 | Firmicutes | Defluviitaleaceae | *uncultured* | Chicken | -3.853 | <0.01 |
| OTU727 | Firmicutes | Ruminococcaceae |  | Chicken | -3.135 | <0.05 |
| OTU741 | Firmicutes | Lachnospiraceae | *Blautia* | Chicken | -3.251 | <0.05 |
| OTU762 | Bacteroidetes | Prevotellaceae | *uncultured* | Chicken | -3.214 | <0.05 |
| OTU8 | Bacteroidetes | Bacteroidaceae | *Bacteroides* | Chicken | -3.212 | <0.05 |
| OTU81 |  |  |  | Chicken | -3.757 | <0.05 |
| OTU192 | Firmicutes | Ruminococcaceae | *uncultured* | Chicken | 4.364 | <0.05 |
| OTU293 | Firmicutes | Christensenellaceae | *uncultured* | Chicken | 3.357 | <0.05 |
| OTU307 | Firmicutes | Ruminococcaceae | *uncultured* | Chicken | 3.105 | <0.05 |
| OTU313 | Firmicutes | Ruminococcaceae | *uncultured* | Chicken | 3.583 | <0.05 |
| OTU477 | Firmicutes | Streptococcaceae | *Streptococcus* | Chicken | 3.266 | <0.05 |
| OTU497 | Firmicutes | Family_XIII | *Incertae_Sedis* | Chicken | 3.594 | <0.05 |
| OTU616 | Firmicutes | Ruminococcaceae | *uncultured* | Chicken | 3.123 | <0.01 |
| OTU628 | Firmicutes | Lactobacillaceae | *Lactobacillus* | Chicken | 4.700 | <0.01 |
| OTU632 | Bacteroidetes | Prevotellaceae | *uncultured* | Chicken | 3.542 | <0.01 |
| OTU659 | Bacteroidetes | S24-7 | *norank* | Chicken | 3.222 | <0.01 |
| OTU70 | Firmicutes | Lachnospiraceae | *Blautia* | Chicken | 3.129 | <0.05 |
| OTU777 | Firmicutes | Erysipelotrichaceae | *Allobaculum* | Chicken | 3.126 | <0.001 |
| OTU93 | Firmicutes | Ruminococcaceae | *uncultured* | Chicken | 3.187 | <0.01 |
| OTU122 | Bacteroidetes | S24-7 | *norank* | Fish | -3.172 | <0.05 |
| OTU136 | Firmicutes | Lactobacillaceae | *Lactobacillus* | Fish | -3.231 | <0.01 |
| OTU182 | Proteobacteria | Enterobacteriaceae | *Escherichia-Shigella* | Fish | -2.864 | <0.05 |
| OTU211 | Firmicutes | Ruminococcaceae | *Oscillibacter* | Fish | -3.048 | <0.05 |
| OTU227 | Bacteroidetes | S24-7 | *norank* | Fish | -3.408 | <0.05 |
| OTU340 | Bacteroidetes | S24-7 | *norank* | Fish | -3.060 | <0.05 |
| OTU356 | Firmicutes | Enterococcaceae | *Enterococcus* | Fish | -2.875 | <0.01 |
| OTU400 | Firmicutes | Ruminococcaceae | *Oscillibacter* | Fish | -3.160 | <0.01 |
| OTU439 | Firmicutes | Ruminococcaceae |  | Fish | -3.382 | <0.001 |
| OTU443 | Firmicutes | Ruminococcaceae | *Incertae_Sedis* | Fish | -3.288 | <0.05 |
| OTU5 | Firmicutes | Lachnospiraceae | *uncultured* | Fish | -3.195 | <0.05 |
| OTU522 | Bacteroidetes | S24-7 | *norank* | Fish | -3.104 | <0.05 |
| OTU531 | Firmicutes | Lachnospiraceae | *Incertae_Sedis* | Fish | -3.045 | <0.05 |
| OTU637 | Firmicutes | Ruminococcaceae | *uncultured* | Fish | -3.129 | <0.001 |
| OTU705 | Bacteroidetes | Rikenellaceae | *RC9_gut_group* | Fish | -3.073 | <0.01 |
| OTU712 | Tenericutes | norank | *norank* | Fish | -3.362 | <0.01 |
| OTU72 | Firmicutes | Defluviitaleaceae | *uncultured* | Fish | -3.714 | <0.01 |
| OTU741 | Firmicutes | Lachnospiraceae | *Blautia* | Fish | -3.300 | <0.05 |
| OTU762 | Bacteroidetes | Prevotellaceae | *uncultured* | Fish | -3.188 | <0.05 |
| OTU8 | Bacteroidetes | Bacteroidaceae | *Bacteroides* | Fish | -3.141 | <0.05 |
| OTU224 | Firmicutes | Staphylococcaceae | *Staphylococcus* | Fish | 3.153 | <0.001 |
| OTU271 | Bacteroidetes | Bacteroidaceae | *Bacteroides* | Fish | 3.060 | <0.01 |
| OTU293 | Firmicutes | Christensenellaceae | *uncultured* | Fish | 3.535 | <0.05 |
| OTU307 | Firmicutes | Ruminococcaceae | *uncultured* | Fish | 2.922 | <0.05 |
| OTU394 | Firmicutes | Erysipelotrichaceae | *Incertae_Sedis* | Fish | 3.806 | <0.05 |
| OTU413 | Bacteroidetes | Prevotellaceae | *Prevotella* | Fish | 3.223 | <0.05 |
| OTU477 | Firmicutes | Streptococcaceae | *Streptococcus* | Fish | 2.746 | <0.05 |
| OTU580 | Firmicutes | Ruminococcaceae | *uncultured* | Fish | 3.367 | <0.05 |
| OTU620 | Firmicutes | Lactobacillaceae | *Lactobacillus* | Fish | 4.625 | <0.05 |
| OTU628 | Firmicutes | Lactobacillaceae | *Lactobacillus* | Fish | 4.560 | <0.001 |
| OTU641 | Firmicutes | Ruminococcaceae | *Ruminococcus* | Fish | 2.823 | <0.01 |
| OTU711 | Bacteroidetes | Porphyromonadaceae | *Parabacteroides* | Fish | 3.050 | <0.05 |
| OTU777 | Firmicutes | Erysipelotrichaceae | *Allobaculum* | Fish | 2.853 | <0.05 |
| OTU91 | Bacteroidetes | Rikenellaceae | *RC9_gut_group* | Fish | 3.005 | <0.05 |
| OTU136 | Firmicutes | Lactobacillaceae | *Lactobacillus* | Pork | -3.301 | <0.001 |
| OTU205 | Firmicutes | Ruminococcaceae | *uncultured* | Pork | -2.916 | <0.05 |
| OTU223 | Firmicutes | Lachnospiraceae | *Roseburia* | Pork | -3.094 | <0.05 |
| OTU271 | Bacteroidetes | Bacteroidaceae | *Bacteroides* | Pork | -3.312 | <0.01 |
| OTU3 | Bacteroidetes | Bacteroidaceae | *Bacteroides* | Pork | -3.617 | <0.01 |
| OTU359 | Proteobacteria | Alcaligenaceae | *Sutterella* | Pork | -2.862 | <0.05 |
| OTU398 | Bacteroidetes | Bacteroidaceae | *Bacteroides* | Pork | -3.020 | <0.01 |
| OTU400 | Firmicutes | Ruminococcaceae | *Oscillibacter* | Pork | -3.094 | <0.05 |
| OTU410 | Bacteroidetes | Prevotellaceae | *Alloprevotella* | Pork | -3.858 | <0.05 |
| OTU439 | Firmicutes | Ruminococcaceae |  | Pork | -3.387 | <0.001 |
| OTU443 | Firmicutes | Ruminococcaceae | *Incertae_Sedis* | Pork | -3.321 | <0.05 |
| OTU5 | Firmicutes | Lachnospiraceae | *uncultured* | Pork | -3.197 | <0.01 |
| OTU522 | Bacteroidetes | S24-7 | *norank* | Pork | -2.933 | <0.05 |
| OTU531 | Firmicutes | Lachnospiraceae | *Incertae_Sedis* | Pork | -2.854 | <0.05 |
| OTU637 | Firmicutes | Ruminococcaceae | *uncultured* | Pork | -3.003 | <0.001 |
| OTU705 | Bacteroidetes | Rikenellaceae | *RC9_gut_group* | Pork | -3.025 | <0.05 |
| OTU741 | Firmicutes | Lachnospiraceae | *Blautia* | Pork | -3.256 | <0.01 |
| OTU8 | Bacteroidetes | Bacteroidaceae | *Bacteroides* | Pork | -3.251 | <0.05 |
| OTU192 | Firmicutes | Ruminococcaceae | *uncultured* | Pork | 4.268 | <0.01 |
| OTU224 | Firmicutes | Staphylococcaceae | *Staphylococcus* | Pork | 3.472 | <0.001 |
| OTU272 | Firmicutes | Lachnospiraceae | *Oribacterium* | Pork | 2.752 | <0.05 |
| OTU274 | Firmicutes | Defluviitaleaceae | *uncultured* | Pork | 2.919 | <0.05 |
| OTU307 | Firmicutes | Ruminococcaceae | *uncultured* | Pork | 2.966 | <0.01 |
| OTU395 | Firmicutes | Lachnospiraceae |  | Pork | 3.321 | <0.05 |
| OTU450 | Firmicutes | Family_XIII | *Mogibacterium* | Pork | 2.772 | <0.05 |
| OTU452 | Firmicutes | Ruminococcaceae |  | Pork | 2.948 | <0.01 |
| OTU477 | Firmicutes | Streptococcaceae | *Streptococcus* | Pork | 2.648 | <0.05 |
| OTU497 | Firmicutes | Family_XIII | *Incertae_Sedis* | Pork | 2.945 | <0.01 |
| OTU51 | Firmicutes | Erysipelotrichaceae | *uncultured* | Pork | 2.881 | <0.05 |
| OTU565 | Tenericutes | norank | *norank* | Pork | 3.191 | <0.01 |
| OTU569 | Firmicutes | Lactobacillaceae | *Lactobacillus* | Pork | 2.996 | <0.05 |
| OTU616 | Firmicutes | Ruminococcaceae | *uncultured* | Pork | 3.446 | <0.05 |
| OTU620 | Firmicutes | Lactobacillaceae | *Lactobacillus* | Pork | 4.798 | <0.01 |
| OTU628 | Firmicutes | Lactobacillaceae | *Lactobacillus* | Pork | 4.081 | <0.01 |
| OTU632 | Bacteroidetes | Prevotellaceae | *uncultured* | Pork | 3.613 | <0.01 |
| OTU659 | Bacteroidetes | S24-7 | *norank* | Pork | 2.970 | <0.01 |
| OTU688 | Firmicutes | Erysipelotrichaceae | *Allobaculum* | Pork | 3.015 | <0.001 |
| OTU777 | Firmicutes | Erysipelotrichaceae | *Allobaculum* | Pork | 3.039 | <0.001 |
| OTU784 | Firmicutes | Ruminococcaceae | *Anaerotruncus* | Pork | 3.657 | <0.01 |
| OTU136 | Firmicutes | Lactobacillaceae | *Lactobacillus* | Soy | -3.180 | <0.001 |
| OTU205 | Firmicutes | Ruminococcaceae | *uncultured* | Soy | -2.913 | <0.01 |
| OTU225 | Tenericutes | norank | *norank* | Soy | -3.657 | <0.05 |
| OTU227 | Bacteroidetes | S24-7 | *norank* | Soy | -3.489 | <0.01 |
| OTU272 | Firmicutes | Lachnospiraceae | *Oribacterium* | Soy | -2.847 | <0.05 |
| OTU292 | Firmicutes | Lactobacillaceae | *Lactobacillus* | Soy | -3.728 | <0.05 |
| OTU305 | Bacteroidetes | S24-7 | *norank* | Soy | -4.345 | <0.001 |
| OTU306 | Firmicutes | Ruminococcaceae | *uncultured* | Soy | -3.348 | <0.05 |
| OTU327 | Bacteroidetes | S24-7 | *norank* | Soy | -2.933 | <0.05 |
| OTU330 | Spirochaetae | Spirochaetaceae | *Treponema* | Soy | -3.704 | <0.05 |
| OTU340 | Bacteroidetes | S24-7 | *norank* | Soy | -3.338 | <0.001 |
| OTU357 | Spirochaetae | Spirochaetaceae | *Treponema* | Soy | -4.121 | <0.01 |
| OTU367 | Firmicutes | Ruminococcaceae | *uncultured* | Soy | -3.127 | <0.05 |
| OTU420 | Firmicutes | Clostridiaceae_1 | *Clostridium_sensu_stricto_1* | Soy | -3.105 | <0.001 |
| OTU439 | Firmicutes | Ruminococcaceae |  | Soy | -3.439 | <0.001 |
| OTU450 | Firmicutes | Family_XIII | *Mogibacterium* | Soy | -2.804 | <0.05 |
| OTU465 | Firmicutes | Ruminococcaceae | *Anaerofilum* | Soy | -3.252 | <0.001 |
| OTU476 | Bacteroidetes | Bacteroidaceae | *Bacteroides* | Soy | -4.674 | <0.001 |
| OTU5 | Firmicutes | Lachnospiraceae | *uncultured* | Soy | -3.215 | <0.01 |
| OTU522 | Bacteroidetes | S24-7 | *norank* | Soy | -3.192 | <0.001 |
| OTU584 | Deferribacteres | Deferribacteraceae | *Mucispirillum* | Soy | -3.082 | <0.001 |
| OTU637 | Firmicutes | Ruminococcaceae | *uncultured* | Soy | -3.091 | <0.01 |
| OTU641 | Firmicutes | Ruminococcaceae | *Ruminococcus* | Soy | -2.633 | <0.05 |
| OTU677 | Firmicutes | Erysipelotrichaceae | *Turicibacter* | Soy | -2.880 | <0.05 |
| OTU705 | Bacteroidetes | Rikenellaceae | *RC9_gut_group* | Soy | -2.989 | <0.01 |
| OTU712 | Tenericutes | norank | *norank* | Soy | -3.367 | <0.05 |
| OTU72 | Firmicutes | Defluviitaleaceae | *uncultured* | Soy | -3.791 | <0.01 |
| OTU741 | Firmicutes | Lachnospiraceae | *Blautia* | Soy | -3.181 | <0.05 |
| OTU762 | Bacteroidetes | Prevotellaceae | *uncultured* | Soy | -3.236 | <0.01 |
| OTU775 | Bacteroidetes | S24-7 | *norank* | Soy | -3.671 | <0.01 |
| OTU81 |  |  |  | Soy | -3.733 | <0.05 |
| OTU122 | Bacteroidetes | S24-7 | *norank* | Soy | 3.000 | <0.01 |
| OTU189 | Bacteroidetes | Bacteroidaceae | *Bacteroides* | Soy | 2.917 | <0.05 |
| OTU21 | Bacteroidetes | S24-7 | *norank* | Soy | 3.112 | <0.01 |
| OTU240 | Bacteroidetes | Prevotellaceae | *uncultured* | Soy | 3.831 | <0.001 |
| OTU271 | Bacteroidetes | Bacteroidaceae | *Bacteroides* | Soy | 3.462 | <0.01 |
| OTU356 | Firmicutes | Enterococcaceae | *Enterococcus* | Soy | 3.604 | <0.05 |
| OTU37 | Bacteroidetes | S24-7 | *norank* | Soy | 3.023 | <0.05 |
| OTU413 | Bacteroidetes | Prevotellaceae | *Prevotella* | Soy | 4.107 | <0.01 |
| OTU473 | Bacteroidetes | S24-7 | *norank* | Soy | 3.061 | <0.05 |
| OTU485 | Proteobacteria | Succinivibrionaceae | *Anaerobiospirillum* | Soy | 3.739 | <0.01 |
| OTU602 | Bacteroidetes | Bacteroidaceae | *Bacteroides* | Soy | 4.785 | <0.001 |
| OTU608 | Bacteroidetes | Prevotellaceae | *Alloprevotella* | Soy | 3.098 | <0.05 |
| OTU609 | Bacteroidetes | S24-7 | *norank* | Soy | 3.386 | <0.05 |
| OTU616 | Firmicutes | Ruminococcaceae | *uncultured* | Soy | 2.848 | <0.01 |
| OTU631 | Firmicutes | Erysipelotrichaceae | *uncultured* | Soy | 3.055 | <0.01 |
| OTU632 | Bacteroidetes | Prevotellaceae | *uncultured* | Soy | 3.518 | <0.001 |
| OTU643 | Bacteroidetes | S24-7 | *norank* | Soy | 3.083 | <0.001 |
| OTU659 | Bacteroidetes | S24-7 | *norank* | Soy | 2.915 | <0.01 |
| OTU697 | Bacteroidetes | Prevotellaceae | *Prevotella* | Soy | 3.546 | <0.01 |
| OTU709 | Bacteroidetes | Rikenellaceae | *RC9_gut_group* | Soy | 2.853 | <0.05 |
| OTU777 | Firmicutes | Erysipelotrichaceae | *Allobaculum* | Soy | 3.516 | <0.001 |
| OTU91 | Bacteroidetes | Rikenellaceae | *RC9_gut_group* | Soy | 3.010 | <0.001 |
