# Supplementary material for: Intake of Meat Proteins Substantially Increased the Relative Abundance of Genus Lactobacillus in Rat Feces
Source: PLoS One. 2016 Apr 4;11(4):e0152678. doi: 10.1371/journal.pone.0152678 (PMC4820228; doi:10.1371/journal.pone.0152678)
Supplement: S6 Table — (DOC) [file pone.0152678.s008.doc]

**S6** **Table Correlation of fecal bacteria with SCFAs concentration.**

|  | Acetic | | Propionic | | Butyric | | Valeric | | Isobutyric | | isovaleric | |
| --- | --- | --- | --- | --- | --- | --- | --- | --- | --- | --- | --- | --- |
|  | Cor | p-value | Cor | p-value | Cor | p-value | Cor | p-value | Cor | p-value | Cor | p-value |
| Alloprevotella |  |  | 0.238 | 0.011 | 0.240 | 0.011 | 0.249 | 0.008 |  |  |  |  |
| Anaerobiospirillum |  |  | 0.215 | 0.023 | 0.328 | 0.001 |  |  |  |  |  |  |
| Bacteroides |  |  | 0.449 | <.0001 |  |  | 0.273 | 0.004 |  |  |  |  |
| Anaerotruncus |  |  |  |  |  |  |  |  |  |  | -0.255 | 0.007 |
| Clostridium_sensu_stricto |  |  |  |  |  |  |  |  |  |  | 0.219 | 0.021 |
| Defluviitaleaceae_uncultured |  |  |  |  |  |  |  |  | -0.249 | 0.008 |  |  |
| Erysipelotrichaceae__incertae_sed |  |  |  |  |  |  | -0.234 | 0.013 |  |  |  |  |
| Lachnospiraceae_ incertae_sedis | -0.208 | 0.027 |  |  |  |  |  |  |  |  | 0.238 | 0.011 |
| Lachnospiraceae_uncultured |  |  |  |  |  |  |  |  | -0.206 | 0.028 |  |  |
| Escherichia_ Shigella |  |  |  |  |  |  |  |  |  |  | 0.227 | 0.017 |
| Prevotella |  |  | 0.279 | 0.003 |  |  |  |  |  |  | 0.193 | 0.040 |
| Fusobacterium | -0.262 | 0.006 |  |  | 0.487 | <.0001 |  |  |  |  |  |  |
| Intestinimonas |  |  |  |  |  |  | 0.252 | 0.007 |  |  |  |  |
| RC9_gut_group |  |  |  |  |  |  | 0.193 | 0.040 |  |  |  |  |
| Ruminococcaceae_unclassified |  |  |  |  |  |  | 0.269 | 0.004 |  |  |  |  |
| Treponema |  |  |  |  | -0.247 | 0.009 |  |  |  |  |  |  |

Note: Kendall tau rank correlation analysis was performed to directly measure the relationships between fecal bacteria at the genus level (only those genera were selected that changed significantly at least in one group compared to casein group) and SCFAs. Cor- Correlation coefficient
